# Carbapenemase-producing *Enterobacteriaceae* circulating in the Reunion Island, a French territory in the Southwest Indian Ocean

**DOI:** 10.1186/s13756-020-0703-3

**Published:** 2020-02-19

**Authors:** Guillaume Miltgen, Pascal Cholley, Daniel Martak, Michelle Thouverez, Paul Seraphin, Alexandre Leclaire, Nicolas Traversier, Bénédicte Roquebert, Marie-Christine Jaffar-Bandjee, Nathalie Lugagne, Céline Ben Cimon, Mahery Ramiandrisoa, Sandrine Picot, Anne Lignereux, Geoffrey Masson, Jérôme Allyn, Nicolas Allou, Patrick Mavingui, Olivier Belmonte, Xavier Bertrand, Didier Hocquet

**Affiliations:** 1 Laboratoire de Bactériologie, CHU Félix Guyon, Allée des Topazes, 97400 Saint-Denis, La Réunion France; 2 UMR Processus Infectieux en Milieu Insulaire Tropical, CNRS 9192, INSERM U1187, IRD 249, Université de La Réunion, Sainte-Clotilde, La Réunion France; 3 Laboratoire d’Hygiène Hospitalière, CHRU Jean Minjoz, Besançon, France; 4 UMR Chrono-Environnement, CNRS 6249, Université de Bourgogne Franche-Comté, Besançon, France; 5 Service d’hygiène hospitalière, CHU Félix Guyon, Saint-Denis, La Réunion France; 6 Laboratoires Réunilab, Sainte-Clotilde, La Réunion France; 7 Laboratoires Cerballiance, Le Port, La Réunion France; 8 Laboratoire de Bactériologie, Groupe Hospitalier Sud Réunion, Saint-Pierre, La Réunion France; 9 Laboratoire de biologie, Centre Hospitalier Gabriel Martin, Saint-Paul, La Réunion France; 10 Laboratoire de biologie, Groupe Hospitalier Est Réunion, Saint-Benoit, La Réunion France; 11 Service de Réanimation polyvalente. Département d’Informatique clinique, CHU Félix Guyon, Saint-Denis, La Réunion France

**Keywords:** Epidemiology, Carbapenemase, French overseas territory, Reunion Island, Indian Ocean, NDM

## Abstract

**Background:**

The spread of carbapenemase-producing *Enterobacteriaceae* (CPE) in the Southwest Indian Ocean area (SIOA) is poorly documented. Reunion Island is a French overseas territory located close to Madagascar and connected with Southern Africa, Indian sub-continent and Europe, with several weekly flights. Here we report the results of the CPE surveillance program in Reunion Island over a six-year period.

**Methods:**

All CPE were collected between January 2011 and December 2016. Demographics and clinical data of the carrier patients were collected. We determined their susceptibility to antimicrobials, identified the carbapenemases and ESBL by PCR and sequencing, and explored their genetic relationship using pulsed-field gel electrophoresis and multi-locus sequence typing.

**Results:**

A total of 61 CPEs isolated from 53 patients were retrieved in 6 public or private laboratories of the island. We found that 69.8% of CPE patients were linked to a foreign country of SIOA and that almost half of CPE cases (47.2%) reached the island through a medical evacuation. The annual number of CPE cases strongly increased over the studied period (one case in 2011 vs. 21 cases in 2016). A proportion of 17.5% of CPE isolates were non-susceptible to colistin. *bla*_NDM_ was the most frequent carbapenemase (79.4%), followed by *bla*_IMI_ (11.1%), and *bla*_IMP-10_ (4.8%). Autochtonous CPE cases (30.2%) harboured CPE isolates belonging to a polyclonal population.

**Conclusions:**

Because the hospital of Reunion Island is the only reference healthcare setting of the SIOA, we can reasonably estimate that its CPE epidemiology reflects that of this area. Mauritius was the main provider of foreign CPE cases (35.5%). We also showed that autochthonous isolates of CPEs are mostly polyclonal, thus unrelated to cross-transmission. This demonstrates the local spread of carbapenemase-encoding genes (i.e. *bla*_NDM_) in a polyclonal bacterial population and raises fears that Reunion Island could contribute to the influx of NDM-carbapenemase producers into the French mainland territory.

## Background

The increasing spread of carbapenemase-producing *Enterobacteriaceae* (CPE) has been witnessed worldwide over the last decade [1–3]. Therapy of infections caused by these extensively drug-resistant bacteria (XDRB) is limited to few options and associated with an increased morbidity and mortality in comparison with those caused by carbapenem-susceptible strains [4–7]. The large population flows from Southern Africa and Indian sub-continent to the Indian Ocean islands can contribute to the dissemination of XDRB in Reunion Island, a French Overseas Territory located close to Madagascar [8, 9]. Hence, the first two CPE isolates detected in Reunion Island were described in 2011 as NDM-1-producing *Klebsiella pneumoniae* and *Salmonella enterica subsp. enterica* (serotype Enteritidis) retrieved from patients previously hospitalized in Mauritius and India, respectively [10]. Likewise, the first OXA-48-like-producing CPE was an OXA-232 produced by *Escherichia coli* found in 2012 in a Mauritian patient who travelled to India [11]. Spread and epidemiology of CPEs have been previously described in Southern Africa, Arabian peninsula and Indian sub-continent [12–18]. However, these CPEs data in the Southwest Indian Ocean area (consisting of Madagascar, Mayotte and Reunion islands, Mauritius, Comoros and Seychelles archipelagos) are poorly known. Albeit in 2015, 0.04% of *E. coli*, 0.19% of *K. pneumoniae*, and 0.48% of *Enterobacter cloacae* isolates were non-susceptible to imipenem in the diagnostic samples in the health care facilities of Reunion Island [19].

Reunion Island has a population of 850,000 inhabitants and has the same health care level as in mainland France. The University Hospital of Reunion Island (UHRI) is the reference hospital in the Southwest Indian Ocean area (SIOA) for the French overseas people but also for the region inhabitants who need specialized care. The Reunion Island, which connects Europe and the SIOA (with five daily flights), could provide the French mainland territory with NDM-carbapenemase producers [20, 21].

Here we report the results of a CPE surveillance program in Reunion Island. The aim of this study was to investigate the CPE spread in Reunion Island between 2011 and 2016 by characterizing the most prevalent resistance determinants and the clonal spread of these XDRB, especially for Reunionese people who have never travelled abroad.

## Methods

### Definitions

We defined a CPE case as a non-redundant patient infected or colonized with a CPE, i.e. a patient who was not previously known as a CPE carrier in one of the laboratories of the surveillance network. An episode of cross-transmission gathered spatiotemporally linked CPE cases (including those sharing the same healthcare workers) who shared a CPE with the same pulsotype (see below). The grouped-cases by episode were defined as the number of cases for an episode of probable cross-transmission i.e. the index case and all the secondary cases. We selected carbapenemase-producers from EUCAST criteria [22]: non-susceptibility to ertapenem (Minimum Inhibitory Concentration, MIC > 0.5 mg/L) and/or to imipenem (MIC > 2 mg/L).

### Study design

Since 2008, the regional antimicrobial resistance surveillance system of Reunion Island collects the microbiological tests performed in six laboratories: two private laboratory groups and four laboratories from public hospitals. These four public hospitals include one bi-site University Hospital (with Northern and Southern sites) and two (Western and Eastern) departmental hospitals with a total about 2500 beds and 250,000 hospital admissions per year. This surveillance system is coordinated by the Federation for Nosocomial Infection Control of Reunion Island (French acronym: FELIN Réunion). The representativeness of this network is estimated to be > 90% of complete-hospitalization days in Reunion Island and 100% of MSO (Medicine Surgery Obstetrical) hospitalizations. In this surveillance system, patients coming from or with a link with a foreign country or Mayotte were systematically screened for multi drug-resistant (MDR) or XDR bacteria by rectal swab within 24 h of admission. In addition, intensive care units (ICUs) and burn units of the UHRI systematically screened patients at their entry and weekly thereafter. Strict contact precautions were maintained when a CPE was identified in the screening sample, and contact patients were identified. Hospitalized contact patients were screened by rectal swabbing for CPE carriage and subjected to contact precautions.

We performed a multicentre retrospective review of all consecutive isolates identified and reported as CPE producer by the Federation for Nosocomial Infection Control of Reunion Island surveillance system between January 2011 and December 2016.

### Patient clinical data

Demographics and clinical data were collected from CPE cases: age, gender, date of admission, date of first positive culture, ward, CPE species, anatomical site of isolation, reason for hospitalization, and comorbidities. A patient was considered as exposed to antimicrobial if he had received an antimicrobial treatment within the 3 months before CPE isolation. The link with a foreign country was defined as a residence, stay or hospitalization abroad (including Mayotte Island) in the year before the hospitalization in Reunion Island. Mainland France was not considered as a foreign country. We retrospectively followed the CPE patients for a maximum of 30 days after the date of the first positive CPE culture to determine all-cause and deaths attributable to CPE infection.

### Bacterial isolates and antimicrobial susceptibility testing

Bacterial species were identified using MALDI-TOF mass spectrometry (Microflex, Bruker Daltonics, Breme, Germany) according to the manufacturer’s recommendations. We evaluated for each isolate the susceptibility to six relevant antimicrobials, which represent the latest therapeutic alternatives. MICs of ertapenem, imipenem, meropenem, tigecycline and fosfomycin were determined using gradient strips (Etest, bioMérieux, Marcy l’Étoile, France). MICs of colistin were determined by broth microdilution method using UMIC colistin kit (Biocentric, Bandol, France). All MICs were interpreted following the 2019 EUCAST recommendations [22]. Isolates with intrinsic resistance to one of the six antimicrobials were excluded from the calculation of the non-susceptibility rate of the corresponding antimicrobial.

### ESBL and carbapenemase identification

Genes encoding ESBLs were identified by PCR and sequencing as previously described [23]. We screened all samples with consensus primers targeting *bla*_CTX − M_, *bla*_SHV_, and *bla*_TEM_ genes. All isolates were evaluated for carbapenemase production by using CARBA-NP test (bioMérieux, La Balme-les-Grottes, France) and GeneXpert system (Cepheid, Sunnyvale, USA) that targets *bla*_KPC_, *bla*_NDM_, *bla*_IMP_, *bla*_VIM_, and *bla*_OXA-48-like_ genes [24, 25]. The presence of carbapenemase-encoding genes (*bla*_KPC_, *bla*_NDM_, *bla*_VIM_, *bla*_IMP_, *bla*_OXA-48-like_, and *bla*_IMI_) was further confirmed by PCR and sequencing for all isolates by the French Associated National Reference Centre for Antibiotic Resistance (FANRCAR, [26]). The *mcr* genes were PCR screened by the FANRCAR in colistin-resistant isolates.

### Molecular genotyping

#### Pulsed field gel electrophoresis (PFGE) analysis

*E. coli*, *K. pneumoniae*, *E. cloacae*, *Citrobacter freundii,* and *Serratia marcescens* isolates were first genotyped by PFGE using *Xba*I (Roche Diagnostics, Meylan, France) as previously described [27]. The Bionumerics software (Applied Math, Kortrijk, Belgium) created a DNA similitude matrix based on calculating the Dice profile for pairwise comparison of strains. The dendrogram was built by using the UPGMA *(Unweighted Pair Group using Arithmetic Averages)* hierarchical algorithm. Pulsotypes were defined according to international recommendations [28]. We applied the threshold of 75% to define the same pulsotype. A PFGE pattern represented by one isolate was called ‘single pulsotype’, while a PFGE pattern shared by ≥2 isolates from several patients was called ‘major pulsotype’. The pulsotypes of all the isolates of the present collection have been determined in the same laboratory, between May and July 2017.

#### Multi-locus sequence typing (MLST) analysis

MLST of *E. coli*, *K. pneumoniae*, *E. cloacae* and *C. freundii* isolates was performed as described elsewhere [29–31]. Hitherto, no MLST scheme exists for *S. marcescens*. Nucleotide sequences were obtained by Sanger sequencing using Applied Biosystems 3500 Genetic Analyser and Sequencing Analysis software 6. Sequence types (STs) were assigned using the MLST databases of the Warwick Medical School for *E. coli* (http://mlst.warwick.ac.uk/mlst/dbs/Ecoli) and of the Institut Pasteur for the other species (http://bigsdb.pasteur.fr/). We defined as a cluster, the isolates sharing the same ST.

### Statistical analysis

Poisson regression analysis was used to determine statistical significance of the time trend in CPE incidence. The chosen significance threshold was 0.05.

## Results

### Demographics and clinical characteristics of CPE cases

**Fig. 1:**
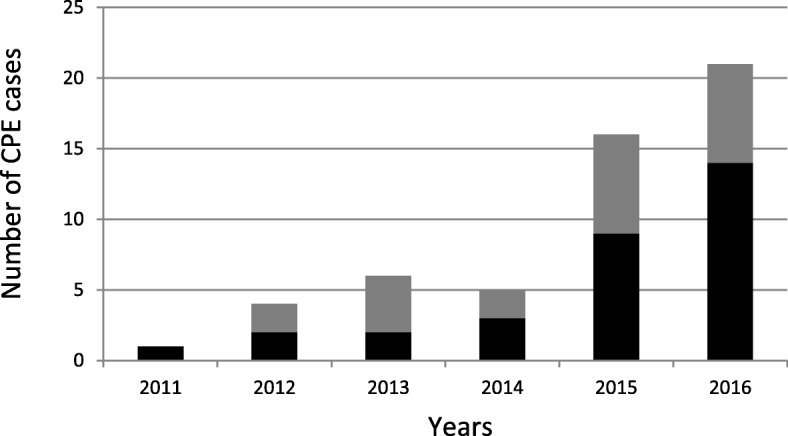
Number of patients with carbapenemase-producing *Enterobacteriaceae* (CPE) detected between 2011 and 2016 in Reunion Island (France). The number of CPE cases linked and not with a foreign country is represented by the black and grey bars, respectively. The upward trends in the ‘total’ [*p* < 0.001], ‘linked with a foreign country’ [*p* < 0.001]’ and ‘not linked with a foreign country’ [*p* = 0.006]’ CPE incidence rates (case per patient-days) were significant

We identified 53 CPE cases in Reunion Island between 2011 and 2016 and observed a dramatic increase over the study period with only one case in 2011 vs. 21 cases in 2016 (Fig. 1). For instance, in the North site of the University Hospital, one CPE was retrieved out of 4147 rectal screenings in 2011 (0.02%) vs. 21 CPE retrieved out of 5063 rectal screenings in 2016 (0.41%). Time trend in CPE incidence was explored using Poisson regression analysis and the CPE incidence rate (total number of CPE cases detected each year) increased significantly on average by 62% per year over the 2011–2016 period (IRR = 1.62; *p* < 0.001). Analysis of clinical data showed that 22 CPE cases (41.5%) had not travelled abroad and that 31 (58.5%) of CPE cases had travelled abroad. Among them, 16 cases (51.6%) had an East origin (i.e. a link with Mauritius or Seychelles islands) and 15 cases (48.4%) a West origin (i.e. a link with Madagascar, Mayotte, or Comoros islands). Mauritius was the most frequent source with 11 CPE cases representing 35.5% of CPE cases who had travelled abroad (20.8% of all CPE cases), followed by Mayotte/Comoros (9 cases, 29%), and Madagascar (6 cases, 19.4%). Over the last 5 years, the proportion of CPE patients who had travelled abroad accounted for 50% in 2012; 33.3% in 2013; 60% in 2014; 56.3% in 2015 and 66.7% in 2016 (Fig. 1).

Table 1 summarizes demographics and clinical data of CPE patients. Male patients accounted for 69.8% of cases and median age was 52. CPE patients were mainly hospitalized in medical ward (50.9%) and ICU (35.9%). Medical evacuation accounted for almost half of all CPE cases (47.2%). The most frequent sites of isolation were the gut flora (73.1%), urine (13.4%), and blood (6%). Treatment data were available for 51 CPE-positive patients. Twelve patients (23.5%) were treated with carbapenems within the 3 months before the CPE detection. Most of the cases (84.9%; *n* = 45) were colonized with CPE and 15.1% (*n* = 8) were infected with CPE. Of the 15 deaths in the cohort, four were attributable to CPE (3 bacteraemias and 1 pulmonary infection). Of the 8 CPE infections, 3 (37.5%) resulted in the death of the infected patients. These 3 deaths were associated with bacteremia. Seven CPE cases (13.2%) resulted from four episodes of cross-transmission that occurred in two medical wards (ICU and burn units).

**Table 1:** Demographic and clinical data of patients with carbapenemase-producing *Enterobacteriaceae* (2011–2016, Reunion Island, France). ^a^ Patients can report multiple isolate sites for each detected CPE. Six patients harboured the same CPE in two isolate sites

Demographics	Number (%) of cases
Gender (*n* = 53)	
Male	37 (69.8)
Female	16 (30.2)
Age (*n* = 53)	
0–18	9 (17)
19–64	28 (52.8)
≥ 65	16 (30.2)
median age	52
Comorbidities (*n* = 53)	
high blood pressure	12 (22.6)
cancer	10 (18.9)
diabetes mellitus	8 (15.1)
obesity	6 (11.3)
ischemic heart disease	5 (9.4)
chronic obstructive pulmonary disease	5 (9.4)
liver cirrhosis	2 (3.8)
Hospital ward (*n* = 53)	
medical	27 (50.9)
ICU	19 (35.9)
surgical	5 (9.4)
other	2 (3.8)
medical evacuation	25 (47.2)
Isolate site (*n* = 67)^a^	
rectal swab / stool	49 (73.1)
urine	9 (13.4)
blood	4 (6)
sputum	3 (4.5)
skin / soft tissue	2 (3)
Exposure to carbapenems (*n* = 51)	
imipenem	9 (17.6)
meropenem	3 (5.9)
no exposure	39 (76.5)
Infections / Colonizations (*n* = 53)	
infection	8 (15.1)
colonization	45 (84.9)
Cross-transmission (*n* = 53)	
yes	7 (13.2)
no	46 (86.8)

### Carbapenemase-producing Enterobacteriaceae (CPE)

We identified 61 CPEs in 53 patients: 47 patients with one CPE, four patients with two CPEs and two patients with three CPEs. One CPE was detected in 2011 (1.6%), five in 2012 (8.2%), six in 2013 (9.8%), six in 2014 (9.8%), 18 in 2015 (29.5%), and 25 in 2016 (41%). The three main species involved were *K. pneumoniae* (*n* = 26; 42.6%), *E. coli* (*n* = 13; 21.3%), and *E. cloacae* (*n* = 9; 14.8%) (Table 2). Over the 6 years of the study, *bla*_NDM_ was the most frequent and represented 79.4% of the carbapenemases, followed by *bla*_IMI_ (11.1%), and *bla*_IMP-10_ (4.8%). The most common *bla*_NDM_ genes were *bla*_NDM-1_ (*n* = 42; 84%) and *bla*_NDM-5_ (*n* = 5; 10%). *bla*_NDM-4_, *bla*_NDM-6_ and *bla*_NDM-7_ genes were detected in one isolate each. *bla*_IMI-1_ gene accounted for 85.7% of *bla*_IMI_ group (six out of seven isolates) and always in *E. cloacae*. One *Enterobacter asburiae* isolate harboured *bla*_IMI-13_ gene. The three isolates that harboured *bla*_IMP-10_ gene were of *S. marcescens*. Half of the CPE isolates (52.5%) also harboured an ESBL-encoding gene with *bla*_CTX-M-15_ being the most common. *bla*_CTX-M-15_ was found in 80.8% of the *K. pneumoniae* CPE isolates, 46.2% of *E. coli* CPE isolates and 16.7% of *C. freundii* CPE isolates. *bla*_SHV-12_ was also detected in two *E. coli* isolates (data not shown).

**Table 2:** Distribution of 61 CPE isolates harbouring 63 carbapenemase-encoding genes (2011–2016, Reunion Island, France). ^a^ Two isolates harboured two carbapenemase-encoding genes: one *K. pneumoniae* isolate harboured *bla*_NDM-1_ and *bla*_OXA-181_, and one *E. coli* isolate harboured *bla*_NDM-1_ and *bla*_VIM-2_

Species	Type of carbapenemase-encoding genes^a^										
	*bla*_NDM_					*bla*_OXA_	*bla*_IMI_		*bla*_IMP_	*bla*_VIM_	Total
	*bla*_*NDM-1*_	*bla*_*NDM-*4_	*bla*_*NDM-5*_	*bla*_*NDM-6*_	*bla*_*NDM-7*_	*bla*_OXA-181_	*bla*_IMI-1_	*bla*_IMI-13_	*bla*_IMP-10_	*bla*_VIM-2_	
*K. pneumoniae*	24		1		1	1^a^					27
*E. coli*	6	1	4	1		1				1^a^	14
*E. cloacae*	3						6				9
*C. freundii*	6										6
*S. marcescens*									3		3
*E. asburiae*								1			1
*E. kobei*	1										1
*P. mirabilis*	1										1
*S. enterica* subsp. *enterica*	1										1
Total	42	1	5	1	1	2	6	1	3	1	63

### Antimicrobial susceptibility testing

Table 3 reports the antimicrobial susceptibilities of the CPE isolates. Overall, 92, 75, and 71% of CPE isolates were not susceptible to ertapenem, meropenem, and imipenem, respectively. We reported 33, 23, and 18% of CPE isolates non-susceptible to tigecycline, fosfomycin, and colistin, respectively. NDM producers were more frequently non-susceptible to meropenem (84%) than to imipenem (66%). All isolates (*n* = 7) that harboured *bla*_IMI_ gene were resistant to colistin. The three isolates harbouring *bla*_IMP-10_ were all resistant to the six antimicrobials tested.

**Table 3:** Antimicrobial susceptibilities of the 61 CPE isolates (2011–2016, Reunion Island, France). ^a^ Two isolates harboured two carbapenemase-encoding genes: one *K. pneumoniae* isolate harboured *bla*_NDM-1_ and *bla*_OXA-181_, and one *E. coli* isolate harboured *bla*_NDM-1_ and *bla*_VIM-2_. ^b^ The clinical breakpoints for imipenem (0.125–4) were used to determine the susceptibility of the isolate of *P. mirabilis.* ^c^ Because of the intrinsic resistance of the *P. mirabilis* species to tigecycline, one isolate of *P. mirabilis* harbouring *bla*_NDM-1_ was excluded for the calculation of non-susceptibility rate of tigecycline. The PK/PD clinical breakpoints for tigecycline (EUCAST 2019; 0.5–0.5) were used to determine the susceptibility of other isolates than *E. coli*. ^d^ Because of the intrinsic resistance of the *S. marcescens* and *P. mirabilis* species to colistin, one isolate of *P.mirabilis* harbouring *bla*_NDM-1_ and 3 isolates of *S.marcescens* harbored *bla*_IMP-10_ were excluded for the calculation of non-susceptibility rate of colistin (N.D.: not determined)

Antimicrobials	Carbapenemase-encoding genes (% of non-susceptible isolates)					MIC Range (mg/L)		MIC_50_ (mg/L)	MIC_90_ (mg/L)
	*bla*_NDM_^a^	*bla*_IMI_	*bla*_IMP-10_	*bla*_OXA-181_	Total	Min.	Max.		
	(*n* = 50)	(*n* = 7)	(*n* = 3)	(*n* = 1)	(*n* = 61)				
Ertapenem	96	71.4	100	0	91.8	0.25	32	8	32
Imipenem	66^b^	100	100	0	70.5^b^	0.5	32	6	32
Meropenem	84	14.3	100	0	75.4	1	32	6	32
Tigecycline	34.7^c^	14.3	100	0	33.3^c^	0.032	24	0.38	6
Colistin	6.1^d^	100	ND^d^	0	17.5^d^	0.06	64	0.25	32
Fosfomycin	22	0	100	0	23	0.125	1024	12	128

### Molecular genotyping

**Fig. 2:**
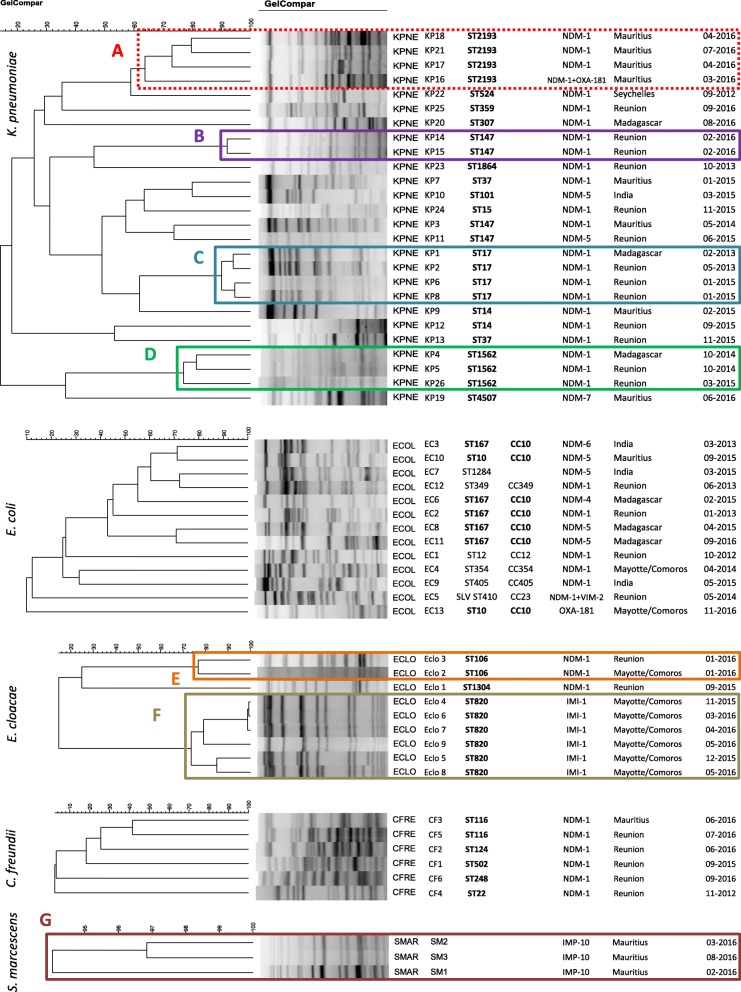
Molecular genotyping (PFGE and MLST analysis) applied to 57 CPE isolates of *K. pneumoniae*, *E. coli*, *E. cloacae*, *C. freundii*, and *S. marcescens* (2011–2016, Reunion Island, France). There is not MLST scheme definition for *S. marcescens*. Data presented: PFGE pattern, bacterial species (KPNE, *K. pneumoniae*; ECOL, *E. coli*; ECLO, *E. cloacae*; CFRE, *C. freundii*, SMAR, *S. marcescens*), sequence type, clonal complex, carbapenemase(s) produced, foreign country visited by the patient within the year before CPE isolation, month and year of CPE isolation. The similarity scales are specific of each bacterial species. The optimization and band-tolerance values used for PFGE dendrogram were 1%

PFGE analysis of the 26 *K. pneumoniae* isolates retrieved one cluster and three major pulsotypes: Cluster A including four ST2193 isolates, pulsotype B including two ST147 isolates, pulsotype C including four ST17 isolates, and pulsotype D including three ST1562 isolates. These 13 isolates harboured both *bla*_NDM-1_ and *bla*_CTX-M-15_; the isolate KP16 (cluster A) co-expressed *bla*_NDM-1_, *bla*_OXA-181_, and *bla*_CTX-M-15_ (Fig. 2). The patients who shared the pulsotypes B and D were hospitalized in the burn unit and those sharing the pulsotype C were hospitalized in the ICU of the Northern University Hospital. The four patients with the *K. pneumoniae* ST2193 (cluster A) came from Mauritius. All *E. coli* isolates displayed different pulsotypes (Fig. 2). Eight STs were identified, with seven isolates belonging to the clonal complex CC10 (ST167, *n* = 5; ST10, *n* = 2). These isolates harboured *bla*_NDM-1_ (*n* = 1), *bla*_NDM-4_ (*n* = 1), *bla*_NDM-5_ (*n* = 3), and *bla*_NDM-6_ (*n* = 1) and *bla*_OXA-181_ (*n* = 1) genes. The nine carbapenemase-producing *E. cloacae* isolates clustered in two major pulsotypes. The pulsotype E (two ST106 isolates with *bla*_NDM-1_) was shared by two patients from the burn unit of Northern University Hospital, while the pulsotype F (six ST820 isolates harbouring *bla*_IMI-1_) came from patients previously hospitalized in Mayotte Island. The six *bla*_NDM-1_ *C. freundii* isolates had unique pulsotypes. In contrast, the three *S. marcescens* isolates belonged to the same pulsotype G, harboured *bla*_IMP-10_, and came from three patients previously hospitalized in the same private clinic in Mauritius.

We found six episodes of cross-transmission. Two of them (*E. cloacae* ST820 – pulsotype F, and *S. marcescens* pulsotype G) gathered patients from the same origin (Mayotte/Comoros and Mauritius, respectively). The other four episodes of cross-transmission of CPE occurred in the Northern University Hospital. The *K. pneumoniae* ST147 (*bla*_NDM-1_; pulsotype B) was shared by two patients with no history of travel abroad. *K. pneumoniae* ST17 (*bla*_NDM-1_; pulsotype C) has been possibly transmitted in ICU from a patient who had travelled in Madagascar (KP1) to three patients who had stayed in Reunion Island (KP2, KP6, KP8). Likewise, *K. pneumoniae* ST1562 (*bla*_NDM-1_; pulsotype D) have been possibly transmitted in the burn unit from a Madagascar-linked patient (KP4) to two patients who had stayed in Reunion Island (KP5, KP26). However, the low percentage of similarity of these three isolates (~ 75%) made it difficult to draw firm conclusions and could suggest an indirect transmission. Similarly, *E. cloacae* ST106 (*bla*_NDM-1_; pulsotype E) has been transmitted in the burn unit from a Mayotte-related patient (Eclo2) to one patient with no history of travel abroad. Overall, we found that 16 out of the 53 CPE cases in Reunion Island were not - directly or indirectly - linked to a foreign country. Since we could have missed less obvious links between CPE carriers and foreign countries, we can consider that a maximum of 30.2% of CPE cases in the Reunion island were autochtonous. In contrast, 37 patients (69.8%) were linked to a foreign country, directly (*n* = 31; with a history of travel abroad) or indirectly (*n* = 6; through the contact with a CPE patient who had travelled abroad).

## Discussion

The global emergence of CPEs is a growing concern and data from the Southern Indian Ocean area are scarce. Here we report the first epidemiological and genotyping analysis of CPEs in an island of this area over a six-year period.

Our study reports an increasing number of CPE cases from 2011 to 2016, in line with the results of surveillance conducted by Santé Publique France institute in France [32]. Among the French regions, Reunion Island has the fourth highest CPE incidence with 3.8 episodes per 100,000 inhabitants over the period 2004–2015 [32]. The origin of CPE carriers differs between Reunion Island and mainland France. Hence, the proportion of CPE cases in mainland France who had travelled abroad was 47% over the period 2004–2015 and decreased overtime. This proportion is higher in the Reunion Island, with 58.5% (31/53) of the CPE cases between 2011 and 2016 who had travelled abroad. This difference most probably results from the growing population flows between the Indian Ocean islands, and the increasing number of medical evacuations towards Reunion Island (125 in 2011 vs. 300 in 2016). Patients repatriated from Mauritius likely had the highest rate of CPE carriage. While this country provides 5% of the medical evacuations, 35.5% (11/31) of CPE cases in Reunion Island with an external origin came from Mauritius (20.8% of all CPE cases).

CPE patients with no history of travel abroad represented 41.5% (*n* = 22) of the CPE cases. Genotyping analysis suggested three episodes of cross-transmissions in the UHRI of isolates recovered from patients who had travelled abroad. Overall, only 30.2% of all CPE cases (*n* = 16) had no direct or indirect (i.e. via a cross-transmission) link with a foreign country. This proportion of cases unrelated abroad is low compared to French national data over the same period (53%). This result is probably linked to the more recent introduction of CPEs on Reunion Island (2011 vs. 2004 in mainland France) and also highlights the strong import pressure to which this French Overseas Territory is subjected.

The most prevalent resistance mechanism was *bla*_NDM_ (79.4%). This epidemiology is radically different from that in mainland France (dominated by *bla*_OXA-48-like_) and reflects the connection between Reunion Island and Indian sub-continent mainly via Mauritius and Seychelles islands. Spread of *bla*_NDM_ in Southern Africa is more complex. NDM-1 carbapenemase was successively described in Kenya and South Africa in 2011, in Tanzania in 2014, and in Madagascar in 2015 [12, 13, 33, 34]. However, tracking *bla*_NDM_ dissemination is difficult because Indian population travel in all these countries directly or via the Indian Ocean islands. Reunion Island has already provided France with NDM producers over the last decade [32] but can become the predominant gateway with the spread of NDM producers in the SIOA. For example, Reunion Island was the second largest supplier of NDM producers in France in the period 2004–2015 (28 isolates for just 850,000 inhabitants), tied with the Provence-Alpes-Côte d’Azur region (28 isolates, 5.1 million inhabitants) and behind the Ile de France region (164 isolates, 12.2 million inhabitants) [32]. The second resistance mechanism was *bla*_IMI_ (11.1%). The proportion of IMI carbapenemase is ten times higher in Reunion than in mainland France [35]. This high prevalence is due to an on-going outbreak of IMI-1 *E. cloacae* in Mayotte Island [36]. The third most prevalent resistance mechanism was *bla*_IMP-10_ (4.8%), harboured by a *S. marcescens* isolates. This proportion is also higher than that reported in mainland France (0.2%) and is presumably due to a nosocomial transmission in a Mauritian clinic [35].

The global rate of resistance to colistin (17.5%) was higher than that reported in the 2016 French National survey (6.8% in CPE) [35]. This high rate was probably due to the chromosomally encoded hetero-resistance to colistin and depending on the PhoP/PhoQ two component regulatory system of the IMI-1 *E. cloacae* strains (cluster XI) which accounted for 9.8% of the isolates [37]. The rate of resistance to colistin in NDM producers (that included isolates of intrinsically resistant *Proteus* sp. or *Serratia* sp.) was similar to national data (8.0% vs. 8.4%). No isolate harboured *mcr* plasmid-mediated resistance genes. The emergence of IMP-10 *S. marcescens* is of concern because the three isolates recovered were non-susceptible to all antimicrobials commonly used to treat CPE infections (Table 3).

Genotypic data (PFGE and MLST) identified 25 CPEs isolated from patients with no history of travel abroad. PFGE analysis suggested that six of them (24%) acquired a CPE isolate from a patient who had travelled abroad (Fig. 2). However, the low percentage of similarity of some isolates (~ 75% for pulsotype D or F) does not allow to assert this transmission with certainty and constitutes a limitation of our study. The high diversity of the genetic background of the CPE isolated from patients with no direct or indirect link with a foreign country suggests multiple transfers of *bla*_NDM_ among local isolates of Reunion Island. On the other hand, *K. pneumoniae* ST147 and *E. coli* ST167 have already been shown to harbour *bla*_NDM_ and could participate in clonal dissemination of this carbapenemase family [38, 39].

## Conclusions

About two-thirds of the patients and the isolates collected in Reunion Island were linked with a foreign country. Therefore, we can reasonably think that the present collection mirrors the epidemiology of CPE of the Indian Ocean area. Besides, most CPE isolates with no link with a foreign country are not clonally related, demonstrating the local spread of carbapenemase-encoding genes (i.e. *bla*_NDM_) in a polyclonal bacterial population.

## Data Availability

The datasets analysed during the current study are available from the corresponding author on reasonable request.

## Abbreviations

CC: Clonal Complex
CPE: Carbapenemase Producing *Enterobacteriaceae*
CTX-M: Cefotaximase-Munich
ESBL: Extended Spectrum Beta Lactamase
EUCAST: European Committee on Antimicrobial Susceptibility Testing
FANRCAR: French Associated National Reference Centre for Antibiotic Resistance
FELIN: Fédération de Lutte contre les Infections Nosocomiales
ICU: Intensive Care Unit
IMI: Imipenemase
IMP: Imipenemase
IRR: Incidence Rate Ratio
MALDI-TOF: Matrix Assisted Laser Desorption Ionisation-Time Of Flight
MDR: Multi Drug Resistant
MIC: Minimum Inhibitory Concentration
MLST: Multi Locus Sequence Typing
MSO: Medicine Surgery Obstetrical
ND: Not Determined
NDM: New Dehli Metallobetalactamase
OXA: Oxacillinase
PCR: Polymerase Chain Reaction
PFGE: Pulsed Field Gel Electrophoresis
SHV: Sulfo Hydryl Variable
SIOA: Southwest Indian Ocean area
ST: Sequence Type
TEM: Temoneira
UHRI: University Hospital of Reunion Island
UPGMA: Unweighted Pair Group using Arithmetic Averages
XDRB: eXtensively Drug Resistant Bacteria
